# Exploring the Implications of Inter-Limb Asymmetries on Sprint, Agility, and Jump Performance in Young Highly-Trained Basketball Athletes: Is There a Relevant Threshold?

**DOI:** 10.3390/medicina60010131

**Published:** 2024-01-10

**Authors:** Fernando Domínguez-Navarro, Javier Gámez-Payá, Borja Ricart-Luna, Iván Chulvi-Medrano

**Affiliations:** 1 Exercise Intervention for Health Research Group (EXINH-RG), Department of Physiotherapy, University of Valencia, 46010 Valencia, Spain; 2 Department of Physiotherapy, Faculty of Health Science, Universidad Europea de Valencia, 46010 Valencia, Spain; 3 Biomechanics & Physiotherapy in Sports Research Group (BIOCAPS), Faculty of Health Sciences, Universidad Europea de Valencia, 46010 Valencia, Spain; 4 Alqueria LAB Department, Valencia Basket Club, 46013 Valencia, Spain; bricart@alqueriadelbasket.com; 5 Department of Physical Education and Sports, University of Valencia, 46010 Valencia, Spain; ivan.chulvi@uv.es

**Keywords:** asymmetry, basketball, countermovement jump, functional assessment

## Abstract

*Background and Objectives*: This study aimed to investigate the magnitude of vertical jump inter-limb asymmetries among young highly-trained basketball athletes and to analyze its impact on sport performance, specifically in sprints, agility, and vertical jumps. *Materials and Methods*: A unilateral countermovement jump (CMJ) was employed to determine Inter-limb Index Asymmetry (IAI) in 320 participants aged from 14 to 18 years, from the Valencia Basket youth academy. IAI was categorized into three groups: 0–9.9%, 10–14.9%, and >15%. The relationship between IAI and performance variables was analyzed through correlation studies (Pearson or Spearman’s). The influence of IAI magnitude was assessed using ANOVA or Kruskal–Wallis analysis, with leg dominance as a covariable. SPSS Statistics version 26 was used for analysis. *Results*: Among all the participants, the mean IAI was 10.6%. Correlation studies revealed non-significant values (*p* < 0.05) between IAI and sport performance variables. The three IAI magnitude groups did not show statistically significant differences in sprint, agility, and jump outcomes. Leg dominance did not seem to influence performance outcomes, except for unilateral CMJ. *Conclusions*: The results obtained challenge the assumption that an IAI above 10% negatively affects sprint, agility, or jump performance in young basketball athletes. Notably, the magnitude of IAI did not influence sport performance parameters, suggesting that the 10–15% threshold from previous studies may not be applicable to this population. The study emphasizes the need to understand lower-limb asymmetries in the context of specific sport task performance, considering the potential evolution over time among affected young athletes.

## 1. Introduction

Functional assessment is a common and crucial procedure in athletes to measure their physical capabilities, performance, and risk of injury. Moreover, this process is even more relevant in young athletes, since they are in the stage of physical development where their physical condition can be most enhanced and where injuries that may compromise their development can be avoided [1,2]. Among the measurements taken, some of the most collected and with the greatest applicability are those related to neuromuscular asymmetries. Particularly, in basketball, due to the succession of primarily unilateral actions, such as jump shots, changes of direction, or pivots, the repetition of these actions [3], together with other anthropometric factors [4], can have repercussions on the player’s development of neuromuscular asymmetries [5].

The measurement of neuromuscular lower limb asymmetries has garnered interest for the last decade. Various sport populations have been assessed [6,7,8], and different methods have been employed, including isokinetic dynamometer [9], running biomechanics [10], sensor of movements analysis [11] and horizontal and vertical jumps [12]. The countermovement jump (CMJ) test, which reflects the muscle function and explosive strength of the lower limb [13], is commonly employed for that purpose [14]. This test has proven to be reliable in detecting functional asymmetries [9], both when performed unilaterally (more robust) and bilaterally (more comprehensive information) [15].

However, in the current literature, there is controversy about the relevance of inter-limb asymmetries in sports performance, as heterogeneity in the results has been observed, varying across factors such as sex, age, sports discipline, or position in the court/role in the team [8,16,17]. Some authors claim it can directly affect sports performance [18,19] and increase the risk of injury [6], while other researchers question this paradigm [20,21]. The magnitude of asymmetry has been suggested to influence the outcomes. Some authors have proposed an inter-limb asymmetry index (IAI) of 10–15% as a threshold to have a relevant impact on performance [17,22], mostly in soccer athletes. However, other researchers have not corroborated this idea [23,24], which yields non-uniform results, leading the issue to a debate that requires further investigation [25]. Moreover, whether these threshold values of relevant asymmetry might be applicable to all sports remains unconfirmed [25].

From another perspective, it is suggested that age may influence the magnitude of asymmetry, although distinct values for relevant threshold asymmetries between collegiate and adult athletes have not been presented. However, it seems logical to assume that the manifestations of asymmetries among youth athletes may differ from those in adults. This variance can be attributed to the occurrence of asymmetry resulting from situations of sport hyperspecialization, a phenomenon not commonly observed during this biological stage [26]. Additionally, the biological immaturity of young athletes may influence neuromuscular adaptations differently compared to adults [27].

With uncertain results regarding the relevance of threshold values for asymmetries related to sports performance, it becomes necessary to analyze these findings in the context of each sport and age group. Specifically, among highly-trained young basketball athletes, where evidence is inconsistent and limited, it is necessary to examine whether the proposed threshold magnitude of 10–15% inter-limb asymmetry, observed in other sports, is also applicable to this population. This study aims to explore the presence of vertical jump inter-limb asymmetries among young highly-trained basketball athletes, to analyze its relevance and impact on sports performance outcomes such as sprints, agility, and vertical jumps, and to analyze the influence of IAI magnitude on the mentioned sports performance outcomes. It is hypothesized that inter-limb asymmetry index values of 10–15% will not serve as a relevant threshold for influencing sports performance among young basketball athletes.

## 2. Materials and Methods

This investigation was a cross-sectional cohort study on a large sample of young highly-trained basketball athletes, carried out in the sports facilities of L’Alqueria del Básquet, Valencia (Spain). This study was approved by the Ethics Committee of the Universitat de Valencia (UV-INV_ETICA-1523453). All experimental procedures followed the statement and recommendations of the Declaration of Helsinki. All included subjects, or legal guardians in the underage cases, received information about the intervention and procedures and signed written informed consent.

### 2.1. Participants

The subjects recruited for the study were members of the male and female youth academy (aged between 14 and 18 years) of Valencia Basket Club, which belongs to the highest tier of both national and European senior and academy categories. The academy athletes adhere to an elite formative methodology, involving 4–5 weekly sessions of collective training, along with personalized technical skills sessions, and 3 weekly sessions dedicated to strengthening and conditioning. To be included in the study, athletes had to: (I) be physically healthy on the day of the evaluation, (II) not have suffered any musculoskeletal injury that would limit sports practice in the 2 months prior to the tests, (III) not present any other physical condition that would prevent them from taking the tests, and (IV) be able to understand the correct performance of the test. The athletes meeting these requirements were invited to participate in the study and were evaluated. The assessments were carried out from 1 October 2022 to 20 October 2022.

### 2.2. Outcomes Assessment

All the testing took place in 3 weeks during the season training period. Before testing, age and anthropometric measurements (height, weight, horizontal and vertical span) were taken to characterize the sample. These variables were collected using a scale and a tape measure, asking the subjects to stand straight and still during the data collection. The sample self-declared their leg dominance or preference after a simple question about their preferent kicking leg. In cases where they were unsure, they were provided with a ball and asked to immediately kick it to determine their dominant leg [28]. Before measuring functional performance outcomes, a standard warm-up was performed, which included 4 min of joint mobility exercises, 4 min of muscle activation exercises, and 4 min of specific basketball movements. Once finished, the athletes performed a functional performance evaluation, following the same order in all cases, based on expected perceived fatigue: bilateral and unilateral CMJ, Cone Drill Test, and the 14–28 m sprint. A familiarization phase was not necessary since, during the season, they repeatedly performed the same evaluation. A member of the research group was responsible for explaining the test procedures and making judgments and corrections regarding proper performance, while another member was tasked with collecting the data provided by the tests. Both researchers, possessing prior experience in conducting these tests, consistently applied the same performance criteria for all participants.

#### 2.2.1. Jump Outcomes: Countermovement Jump (CMJ)

This test was evaluated in all athletes; first, bilaterally, and, later, unilaterally. For bilateral execution, participants were placed in the center of the platform in a comfortable standing position. For unilateral CMJ, participants stood unilaterally on the leg to be evaluated, with the raised leg placed in a position of 90° of hip and knee flexion. Since body tucking was not used, participants were instructed to adhere to the following instructions to standardize their performance. The participants were instructed to squat to a depth (90°). For this purpose, a previous measurement was made with an analog goniometer to establish the 90° of knee flexion. To provide kinesthetic feedback, an elastic band was adjusted so that when performing the downward phase, the bottom part would perceive the elastic band and could perform the upward phase. Participants were instructed to shorten the duration between the lowering and ascending stages of the jump, to jump as high as they could, and to keep their hands on their hips throughout the entire exercise. In the case of unilateral CMJ, swinging with the leg raised was prohibited. The athletes, starting from a position of maximum knee extension, perform a vertical jump, as high as possible, after previously performing a knee flexion to gain momentum. The acceptable technique included jumping vertically without body tucking or bending the knees or hips during jumping. Otherwise, the jump vas considered invalid. Participants were instructed to employ their own landing strategy without any specific kinematic instructions and to land on the same spot where they initially stood before the jump. The examiner counted down “3, 2, 1” while participants had to remain in a standing position and the examiner shouted “Jump!” for jump initiation. To measure the height of the jump, the Optojump jump platform (Microgate, Bolzano, Italy) [29] was used; all athletes performed 3 jumps bilaterally and 3 unilaterally, allowing 30 s of recovery between each jump. The highest attained height in the vertical jump, just immediately before the descent jump phase, was recorded, and used for subsequent analysis, expressed in centimeters. The CMJ has demonstrated high reliability values both for bilateral (ICC = 0.95) [30] and unilateral jump (ICC = 0.89) [15]. Bilateral CMJ was used as performance outcomes, while unilateral CMJ values were used to calculate IAI, by applying the following formula: [(dominant limb–non-dominant limb)/dominant limb] × 100, where the dominant limb was the one with the highest jump height [31].

#### 2.2.2. Agility Outcome: Cone Drill Test

For this test, athletes were asked to run a circuit focused on the outcome of different changes of direction, in which they had to touch the 4 cones in the minimum possible time [32]. The time taken to complete the circuit was measured. Two attempts were made per athlete, the best record was used for further analysis.

#### 2.2.3. Sprint Outcome: 14–28 m Sprint

The 14–28 m sprint variables (minimum possible time in running 14 and 28 m, respectively) were also considered as measures of sports performance. These distances were considered due to their specificity in basketball practice, corresponding to half-court and full-court distances, respectively. Three photocells were placed to record the athlete’s time over 14 m and 28 m. Each athlete had two attempts at 28 m. The best record at 14 and 28 m was considered for later analysis [33]. The equipment used was Witty Photocells (Microgate Srl; Bolzano, Italy), which have proven to be sensitive and reliable (0.001-s accuracy) [33].

### 2.3. Data Analysis

First, a descriptive analysis of the total sample was performed for all outcomes, including mean values, standard error of the mean (SEM), and percentages when appropriate. The normal distribution of data was assessed using Kolmogorov–Smirnov test. Based on the literature regarding the significance values of IAI [17,22] and following the methodology employed in previous studies [23], participants were divided into 3 groups based on the presented IAI: non-relevant asymmetry (IAI of 0–9.9%), relevant smaller asymmetry (10–14.9) and relevant larger asymmetry (>15%). Aligned with the study’s objectives, the influence of inter-limb asymmetry on performance outcomes (sprint-agility and jumping) was analyzed. Initially, correlation analyses (either Pearson’s or Spearman’s) were conducted to explore the relationship between inter-limb asymmetry and performance outcomes for both males and females. Subsequently, to delve into the influence of different degrees of asymmetry, between-group comparison analyses (either ANOVA or Kruskal–Wallis) were carried out for both sexes. Post hoc comparisons between groups, with Bonferroni corrections, were conducted if significant differences were observed. The dominant leg was evaluated as a covariable. Confidence intervals were 95%. Statistical significance was set at 0.05. All statistical analyses were conducted using IBM SPSS version 26 (Armonk, New York, NY, USA).

## 3. Results

A total of 320 athletes were evaluated, including 165 women (51.6%) and 155 men (48.4%), with a mean age of 15.83 years (SEM = 1.4). Anthropometric information is detailed in Table 1. The mean asymmetry index was 10.80% (SEM = 6.5) for men and 10.43% (SEM = 7.1) for women. However, the proportion of athletes with IAI above 10% was 38.7% (n = 60) in men and 40% (n = 65) in women. Leg dominance exhibited similar values among men (77.4% right-leg dominant) and women (82.4% right-leg dominant).

All correlation analyses, including Pearson’s for parametric variables (men CMJ bilateral, men 14 m Sprint, men 28 m Sprint, men Cone Drill Test, women CMJ bilateral, women CMJ right, women CMJ left) and Spearman’s for non-parametric variables (men CMJ right, men CMJ left, women 14 m Sprint, women 28 m Sprint, women Cone Drill Test) revealed non-significant values (*p* > 0.05) between IAI and performance outcomes for both male and female (Table 2).

Subsequently, between-group comparison analyses (ANOVA and Kruskal–Wallis) were conducted among the three IAI groups. Once again, non-significant differences were found both for males and females in all evaluated performance outcomes (Table 3), indicating no influence for magnitude of IAI in performance outcomes. The dominant leg was found to significantly influence IAI in women (*p* < 0.001), but not in men; while also significant influence was observed in unilateral CMJ values (men: CMJ left *p* = 0.026, F = 3.719; women: CMJ right *p* = 0.002, F = 5.353). The rest of the performance outcomes were not influenced by leg dominance.

## 4. Discussion

The present study aimed to investigate vertical jump inter-limb asymmetries in a cohort of young highly-trained basketball athletes, as well as to analyze their impact and relevance on specific sport performance outcomes such as sprints, agility, and jumps. Following the analysis of 320 young basketball athletes, the Inter-limb Asymmetry Index, measured with unilateral CMJ, did not show a relevant impact on performance-related outcomes. No significant results were found in correlation studies or the comparison of asymmetric magnitude across groups, for both males and females. Furthermore, within the present sample, approximately 40% of them exhibited an IAI above 10%, with a mean total index asymmetry score of 10.6%, which is a higher percentage than found in some studies [6,10], but similar to others [16]. These findings suggest that the so-called “relevant asymmetries”, referring to inter-limb asymmetries values higher than 10–15%, are frequent in young basketball athletes, without, apparently, negatively impacting sports performance outcomes. Therefore, this supports the hypothesis that the proposed asymmetry threshold of values exceeding 10%, suggested to significantly impact performance in soccer [8,12], may not apply to basketball athletes. Moreover, these findings align with those obtained by Bell et al. [23] and Lockie et al. [24], indicating that values exceeding 10% asymmetry were not associated with worse performance outcomes.

The relevance and impact of IAI on sports performance and risk of injury is an intriguing topic in recent years. Existing literature suggests that asymmetry significantly impacts performance at levels above 10% or 15% of IAI [17,22], while asymmetry below 10% is considered non-relevant. Following this assumption, participants in the present study were grouped into three categories based on the magnitude of the asymmetry exhibited: no relevant asymmetry (IAI of 0–9.9%), small asymmetry (IAI of 10–15%) or larger asymmetry (IAI > 15%). However, the between-group analysis comparison revealed that these potentially relevant thresholds might not apply to the present sample, as non-significant differences in sprint, vertical jump, and agility were observed based on the magnitude of IAI, questioning its significance. Isin et al. [34] found similar results to ours in male youth soccer athletes, observing no differences for sprint or change of direction when comparing different magnitudes of vertical jump inter-limb asymmetry. Similarly, Bell et al. [23] did not observe worsening vertical performance when comparing the magnitude of asymmetry divided into four groups (IAI of 0–5%, 5–10%, 10–15%, and >15%). Additionally, Lockie et al. [24] divided the sample into lesser and larger asymmetry based on vertical jumping, observing larger asymmetry did not negatively impact speed or agility. Conversely, contrasting results emerge from other studies that did find a significant correlation between inter-limb asymmetries and sprint/agility in soccer athletes. This is evident in the studies by Bishop et al., where asymmetry was found to be relevant whether analyzed through vertical drop [35] or horizontal jumps [12], as well as in Michailidi’s study [36], which compared the dominant with the non-dominant leg jumps.

The divergence in results underscores the necessity to comprehend the complexity of the relationship between asymmetries and performance, considering both the sport-specific task and participants’ contexts, while also considering the methodological characteristics of the studies. Many of the referenced studies employed limited sample sizes, particularly in groups of larger asymmetries when subgrouping the sample [23,34]. Additionally, there is notable heterogeneity in the methods used to analyze asymmetry, as well as in the demographic and sports-related characteristics of the evaluated samples. Since different factors such as sex [8], age of athletes [17], sports discipline [37], position in the court [11], and level of performance (amateur or elite) [6] seem to influence the degree of lower-limb asymmetry, it is crucial to consider these factors when interpreting and comparing results across studies.

Within various sports disciplines, the physiological and functional demands differ, potentially influencing the development of inter-limb asymmetry [38]. In the context of basketball, the prevalence of asymmetrical actions and unilateral movements in its practice may contribute to the emergence of greater asymmetries compared to other sports [39]. In our study, the observed higher IAI in basketball athletes supports this notion, exceeding reported values in soccer and futsal. Specifically, female soccer athletes displayed an IAI of 8.45% [35], while male soccer and futsal athletes exhibited values of 8.37% and 7.93%, respectively [6]. Additionally, lower IAI values (5–6%) were obtained in the study by Dos ‘Santos et al. [28] within a collegiate multisport sample. Aligning with our IAI results, Fort-Vanmeerhaeghe et al. [16] found vertical jump asymmetries of 10–15% in basketball and volleyball athletes, emphasizing similar asymmetric jump patterns in both sports. Additionally, Mendonça et al. [37] found inter-limb asymmetries to be higher in basketball than in soccer athletes, also consistent with other studies [7]. Thus, since the physiological demands and degree of asymmetry are different in various sports, it seems logical to infer that there cannot be a single relevant asymmetry threshold value common for all sports, and the relevance for specific magnitude asymmetries should be considered. Furthermore, concerning the relationship between asymmetries and sports injury, in basketball, this relation is not fully supported. In the Guerra et al. [11] study, motion sensors were employed to capture asymmetries related to balance and changes in direction. This study found that these asymmetries were not correlated with having suffered a previous injury. Observing these findings, it is suggested that asymmetries might be an adaptative response that an athlete found to restore a dynamic stable support to achieve an efficient performance, and might not be disruptive.

Age is also a determining factor for the presence of asymmetries, as varying degrees have been observed between young and senior athletes [17]. Asymmetries are adaptative to the sports context and physiological maturation stage, fluctuating as the subjects mature and their physical capacities, such as balance, strength, or mobility, evolve [40]. Therefore, it should be of relevance to our study, given that the sample consists of athletes aged between 14 and 18, so it could be assumed that physiological maturity is not yet complete for some of them, and asymmetries may fluctuate across time. However, predicting how asymmetry evolves is not straightforward, as it depends on the intensity and duration of practice and competition level. [26]. Some authors suggest that IAI may decrease over time [26], with athletes tending to equalize their inter-limb strength capacities. Conversely, others propose that asymmetries are more prominent in more experienced athletes [41].

In the present study, IAI was calculated as the percent difference between the higher and the lower performance leg in the uniliteral CMJ. However, results revealed that higher leg performance did not always correlate with the dominant leg. Concretely, leg dominance only appeared to be relevant for unilateral CMJ values, but not for the rest of the outcomes. This fact has also been exposed by other authors, suggesting a low correlation between the subjective self-perception of leg dominance and the objective higher leg performance [16,32]. The analysis of asymmetries through unilateral CMJ has been employed in various similar studies, while others have used strength values obtained by isokinetic dynamometer [9,17]. Although there is no solid consensus regarding the most reliable method to calculate inter-limb asymmetries, isokinetic strength values have been criticized for their lesser functional transfer to sports activities and the lack of uniformity in the angle used to evaluate strength, which influences the obtained results [42]. However, some studies have revealed that functional testing, such as jumping, could be as reliable as isokinetic performance [43], with the advantage of being easier to implement and requiring less expensive equipment, which may not be available for all coaches and practitioners. Additionally, when considering adolescents, functional movements, like CMJ, appear to be less influenced by the offset in maturation than muscular strength. Consequently, functional tests may be considered an option with fewer biases by the maturation stage [44].

Measuring lower-limb asymmetries is commonly performed by strength and conditioning coaches and physiotherapists as part of the functional evaluation of athletes. While it may guide the development of both performance enhancement and injury prevention/reduction training programs, the impact of these asymmetries on performance outcomes is not yet clear. However, this does not negate its usefulness, as the obtained results should always be understood within the athletic, maturational, and anthropometric context of the athlete. Integrating these findings along with other physical–functional, psychological, and maturation aspects can provide a more comprehensive view of the athlete’s potential performance.

### Strengths, Limitations, and Future Research

The main strength of this research is that it was carried out on a large sample (320 participants) of young highly-trained athletes. Moreover, three validated tests of sports performance related to relevant actions in basketball (agility, sprinting, and jumping) were evaluated. Thus, it offers a complete and specific view of performance in basketball.

However, there exist some limitations to be mentioned. First, subclassifying the sample according to the magnitude of observed asymmetry resulted in non-homogeneous groups in terms of the number of subjects. Likewise, the cohort nature of the study allows us to know the degree of asymmetry only at a specific moment in time, and not how it evolves. Additionally, in this research, the maturational age of the participants was not taken into account. Considering that athletes of these ages are in a developmental phase, where rapid and remarkable changes in physical status and function occur, for future research, the maturational status could be collected to analyze the influence of the point of maturity on the development of neuromuscular asymmetries.

## 5. Conclusions

The results obtained allow the suggestion that the assumption that an inter-limb asymmetry value above 10% negatively affects sprint, agility, or jump performance does not apply to young basketball athletes. Notably, the magnitude of IAI did not influence sports performance parameters, suggesting that the 10–15% threshold from previous studies may not be applicable to this population. The study emphasizes the need to understand lower-limb asymmetries in the context of specific sports task performance, considering potential evolution over time among young athletes influenced by various factors.

## Figures and Tables

**Table 1:** Demographic, anthropometric and dominant-leg outcomes for total sample and index asymmetry groups.

	Total Sample (n = 320)	0–9.9% IAI Group (n = 194)	10–14.9% IAI Group (n = 57)	>15% IAI Group (n = 69)
**Demographic outcomes**				
Age (years)	15.83 (1.4)	15.50 (1.7)	16.22 (1.4)	16.11 (1.1)
Men (n)	155 (48.4%)	95 (61.3%)	27 (17.4%)	33 (21.3%)
Women (n)	165 (51.6%)	99 (60.0%)	30 (17.6%)	36 (21.8%)
**Anthropometric outcomes**				
Men				
Height (cm)	196.67 (11.9)	193.22 (38.9)	190.67 (5.1)	201.44 (22.1)
Weight (kg)	103.80 (11.6)	107.13 (15.0)	98.30 (9.4)	105.11 (20.6)
Horizontal span (cm)	191.31 (13.0)	190.11 (12.3)	187.20 (12.6)	194.56 (14.0)
Vertical span (cm)	244.11 (14.7)	240.35 (14.7)	238.15 (15.0)	248.30 (16.2)
Women				
Height (cm)	175.75 (11.3)	174.79 (17.4)	176.51 (7.8)	177.76 (8.6)
Weight (kg)	68.70 (7.2)	67.78 (7.1)	67.98 (10.6)	71.84 (12.1)
Horizontal span (cm)	175.23 (9.3)	174.62 (9.0)	174.11 (9.9)	177.0 (9.9)
Vertical span (cm)	219.43 (12.6)	218.15 (12.0)	219.06 (13.0)	220.11 (12.8)
**Dominant leg (n (%))**				
Men				
Right (n)	120 (77.4%)	71 (59.2%)	23 (19.2%)	26 (21.6%)
Left (n)	35 (22.6%)	24 (72.7%)	5 (15.1%)	6 (18.2%)
Asymmetry index (%)	10.80 (6.5)	5.77 (2.0)	12.53 (1.6)	18.54 (3.6)
Women				
Right (n (%))	136 (82.4%)	77 (56.6%)	25 (18.4%)	34 (25%)
Left (n (%))	31 (17.6%)	22 (71.0%)	6 (19.3%)	3 (9.7%)
Asymmetry index (%)	10.43 (7.1)	5.36 (1.6)	12.40 (1.8)	22.8 (3.8)

IAI: Inter-limb Asymmetry Index.

**Table 2:** Correlation analysis (Pearson’s and Spearman) between Inter-limb Asymmetry Index and performance outcomes. Values are expressed in correlation coefficient (*p* values).

	CMJ Bilateral	CMJ Right	CMJ Left	14 m Sprint	28 m Sprint	Cone Drill Test
Men (n = 155)						
IAI	−0.054 (0.505)	−0.106 (0.187) ^1^	−0.032 (0.690) ^1^	−0.068 (0.396)	−0.091 (0.257)	0.010 (0.902)
Women (n = 165)						
IAI	0.030 (0.706)	−0.141 (0.073)	0.055 (0.483)	0.067 (0.395) ^1^	0.093 (0.237) ^1^	0.021 (0.788) ^1^

^1^ indicates non-parametric analysis (Kruskal–Wallis) for non-normal distribution variables. CMJ: Countermovement Jump. IAI: Inter-limb Asymmetry Index.

**Table 3:** Comparison outcomes for the index asymmetry groups for males and females, with leg-dominance as a covariable.

	0–9.9% IAI Group	10–14.9% IAI Group	>15% IAI Group	Between-Group Comparison	Leg Dominance (Covariable)
				(*p* Values, F)	(*p* Values, F)
Men					
Subjects (n(%))	95 (61.29)	27 (17.42)	33 (21.29)		
Index Asymmetry (%)	5.77 (2.0)	12.53 (1.6)	18.54 (3.6)	<0.001 * (124.53)	0.003 * (8.971)
CMJ bilateral (cm)	39.06 (5.3)	39.43 (6.6)	37.75 (6.1)	0.643 (0.443)	0.136 (1.428)
CMJ right	22.23 (3.3)	21.91 (4.3)	21.14 (5.3)	0.101 ^1^ (0.490)	0.595 (0.633)
CMJ left	22.29 (3.4)	22.42 (4.8)	22.07 (5.6)	0.800 ^1^ (0.460)	0.026 * (3.179)
14 m Sprint (s)	2.38 (0.1)	2.40 (0.2)	2.34 (0.2)	0.366 (1.013)	0.555(0.697)
28 m Sprint (s)	4.17 (0.2)	4.20 (0.3)	4.11 (0.3)	0.221 (1.524)	0.367 (1.063)
Cone Drill Test (s)	5.75 (0.5)	5.81 (0.4)	5.76 (0.5)	0.807 (0.443)	0.634 (0.572)
Women					
Subjects (n(%))	99 (60)	30 (17.57)	36 (21.81)		
Index Asymmetry (%)	5.36 (1.6)	12.40 (1.8)	22.8 (3.8)	<0.001 * (112.450)	<0.001 * (135.279)
CMJ bilateral	39.06 (5.3)	39.43 (6.6)	37.75 (6.1)	0.871 (0.138)	0.631 (0.577)
CMJ right	16.80 (2.6)	16.21 (3.5)	15.73 (4.1)	0.707 (0.347)	0.002 * (5.353)
CMJ left	16.79 (2.7)	16.7 (2.8)	17.25 (3.7)	0.200 (1.626)	0.844 (0.274)
14 m Sprint	2.38 (0.1)	2.40 (0.2)	2.34 (0.2)	0.823 ^1^ (0.389)	0.782 (0.359)
28 m Sprint	4.17 (0.2)	4.20 (0.3)	4.11 (0.3)	0.579 ^1^ (1.093)	0.146 (1.818)
Cone Drill Test	5.75 (0.5)	5.81 (0.4)	5.76 (0.5)	0.693 ^1^ (0.733)	0.150 (1.794)

^1^ indicates non-parametric analysis (Kruskal–Wallis) for non-normal distribution variables. * indicates significant (*p* < 0.05) differences. CMJ: Countermovement Jump.

## Data Availability

Data will be available under reasonable request to the corresponding author.
